# Bioremoval of Co(II) by a novel halotolerant microalgae *Dunaliella* sp. FACHB-558 from saltwater

**DOI:** 10.3389/fmicb.2024.1256814

**Published:** 2024-04-30

**Authors:** Chenglong Liu, Xueer Wen, Huiqiao Pan, Ying Luo, Junyang Zhou, Yuzhe Wu, Zhiyong Zeng, Ting Sun, Jun Chen, Zhangli Hu, Sulin Lou, Hui Li

**Affiliations:** ^1^Guangdong Engineering Research Center for Marine Algal Biotechnology, Guangdong Provincial Key Laboratory for Plant Epigenetics, Shenzhen Key Laboratory of Marine Bioresource and Eco-Environmental Science, Longhua Innovation Institute for Biotechnology, College of Life Sciences and Oceanography, Shenzhen University, Shenzhen, China; ^2^Key Laboratory of Optoelectronic Devices and Systems of Ministry of Education and Guangdong Province, College of Optoelectronic Engineering, Shenzhen University, Shenzhen, China; ^3^Department of Embryology, Carnegie Institution for Science, Baltimore, MD, United States

**Keywords:** biosorbent, cobalt, *Dunaliella*, environmental pollution, heavy metal, microalgae

## Abstract

Cobalt pollution is harmful to both the aquatic ecosystem and human health. As the primary producer of aquatic ecosystems in hypersaline environments, unicellular planktonic *Dunaliella* microalgae is considered to be a low-energy and eco-friendly biosorbent that removes excess cobalt and enhances the vitality of coastal and marine ecosystems. In this study, we found that the halotolerant microalga named *Dunaliella* sp. FACHB-558 could grow under a salinity condition with 0.5–4.5 M NaCl. A phylogenetic analysis based on the *rbc*L gene revealed that *Dunaliella* sp. FACHB-558 is a close relative of *Dunaliella primolecta* TS-3. At lab-scale culture, *Dunaliella* sp. FACHB-558 exhibited high tolerance to heavy metal stresses, including cobalt, nickel, and cadmium. Treatment with 60 μM cobalt delayed its stationary phase but ultimately led to a higher population density. Furthermore, *Dunaliella* sp. FACHB-558 has the ability to adsorb the cobalt ions in the aquatic environment, which was evidenced by the decreased amount of cobalt in the culture medium. In addition, the tolerance of *Dunaliella* sp. FACHB-558 to cobalt stress was correlated with enhanced nitric oxide content and peroxidase activity. The autophagy inhibitor 3-MA enhanced nitric oxide burst, increased peroxidase activity, and accelerated the bioremoval of cobalt, suggesting that the autophagy pathway played a negative role in response to cobalt stress in *Dunaliella* sp. FACHB-558. In summary, our study identified a novel microalga possessing high cobalt tolerance and provided a promising natural biosorbent for the research and application of heavy metal bioremediation technology.

## 1 Introduction

Unlike organic pollutants, heavy metals cannot be decomposed and can gradually accumulate in organisms through the food chain. This accumulation can ultimately lead to the heavy metals being stored in the human body after continuous enrichment (Lambert and Davy, 2011). Cobalt is an essential micronutrient for humans, animals, crops, and algae (Lavoie et al., 2012). However, excessive cobalt from sewage irrigation, sludge application, pesticides, and fertilizers will cause agricultural disasters (Haddad et al., 2017). Consequently, cobalt-contaminated water or food will cause serious health problems such as low blood pressure, paralysis, diarrhea, and bone defects (Eid et al., 1958; Greig and Macleod, 1977; Nicoloff et al., 2006; Ignjatović et al., 2013). Nuclear wastewater contains radioactive elements, such as cobalt-60, which are easily absorbed by organisms in marine sediments and may affect the marine environment for a long time (Jia et al., 2018). Due to its potential toxicity, cobalt contamination has become a serious environmental problem. Remediation of cobalt pollution in the aquatic environment helps to enhance the vitality of the aquatic ecosystem and has become a sustainable development goal (Jiang et al., 2022).

In recent years, the biosorption of natural substances derived from bacteria, fungi, yeasts, algae, and other biological sources to control and remove heavy metal pollution has been widely discussed. Bioremoval technology has been a hotspot in the field of heavy metal pollution control, because of its potential application prospects (Ayangbenro and Babalola, 2017). Biosorbents at low concentrations can efficiently and rapidly separate metal ions from the solvent, thereby reducing the concentration of heavy metal ions in the solution. It is an ideal choice for the treatment of wastewater with a large capacity (Bulgariu and Bulgariu, 2018; Tripathi et al., 2023). Microalgae have the potential to passively adsorb heavy metals or other pollutants onto their extracellular structures. They serve as low-cost and environmentally friendly natural biosorbents (Pleissner and Smetana, 2022). In the past few decades, a number of highly efficient alga sorbents have been identified (Thongpitak et al., 2019; Aragaw and Bogale, 2021; Šimonovičová et al., 2021). A biosorption study combined with a FTIR assay showed *Synechocystis pevalekii* and *Scenedesmus bernardii* can be used as biosorbents to remove cobalt ions (Fawzy et al., 2020). In addition, under ideal laboratory conditions, *Phormidium tenue* and *Chlorella vulgaris* collected from the Wadi Hanifah stream in the Kingdom of Saudi Arabia can remove 94 and 87% of cobalt, respectively (Abdel-Raouf et al., 2022). Their broad application prospects as biosorbents for effectively absorbing cobalt ions in aquatic conditions have been widely discussed (Abdel-Raouf et al., 2022). *Tetraselmis* sp. had the potential to be an outstanding heavy metal accumulator and biodiesel producer (Dammak et al., 2022). Phycoremediation of heavy metals from contaminated water by oleaginous microalgae is an eco-friendly and emerging trend.

In the bioremediation of large-scale heavy metal contamination in the environment, natural biosorption is not considered competitive. Attempts to commercialize natural biosorption have not been successful thus far (Aragaw and Bogale, 2021). Marine algae have shown potential as an effective natural biosorbent for heavy metals. With many advantages, such as minimal growth requirements, marine algae can be easily applied in large-scale commercial use. However, the extra cost of algae harvesting needs to be considered. Therefore, cultivating low-cost natural microalgae sorbents with high tolerance to heavy metals has become an urgent task and prerequisite for the development of bioremediation technology addressing heavy metal pollution in aquatic environments (Rajfur, 2013; Fomina and Gadd, 2014).

Marine microalgae widely existing in the ocean with a fast growth rate and large-scale cultivation ability have the potential adsorption capacity to reduce the content of heavy metals in the marine ecosystem. The genus *Dunaliella* consists of a class of halophilic unicellular planktonic algae with a wide range of salinity tolerance and is widely distributed in the ocean, salt lakes, and wetlands around the world (Ramos et al., 2011). *Dunaliella* spp. can normally survive in an aquatic environment with NaCl concentrations ranging from 0.5 to 5.5 M (Lou et al., 2020). Studies have shown that *Dunaliella tertiolecta* has great potential to repair Zn^2+^ pollution in the water (Tsuji et al., 2003). Thus, the genus *Dunaliella*, with its unique marine adaptability and robust heavy metal tolerance, can be a potential candidate for bioremediation of marine heavy metal pollution. However, the diversity of cobalt adsorption capacity in different *Dunaliella* species is not clear, and the corresponding molecular mechanism of cobalt stress tolerance is still unknown.

In this study, we characterized a novel *Dunaliella* species, *Dunaliella* sp. FACHB-558, with high cobalt biosorption capacity, and investigated the molecular mechanism underlying its high tolerance to severe cobalt stress. This study provided insights into the mining of *Dunaliella* resources for heavy metal responses and the identification of natural biosorbents suitable for coastal areas and oceans. Moreover, this study expands the pool of potential natural biosorbents for heavy metal bioremediation and helps the understanding of how microalgae cope with heavy metal stress, which can guide future research and the development of more sustainable, low-cost, and large-scale bioremediation strategies for commercial application.

## 2 Materials and methods

### 2.1 Strain and cultivation conditions

The *Dunaliella* sp. FACHB-558 was obtained from the Freshwater Algae Culture Collection at the Institute of Hydrobiology, Chinese Academy of Sciences.^1^ The *Dunaliella salina* CCAP 19/18 was obtained from the Culture Collection of Algae and Protozoa (CCAP).^2^ *Dunaliella* cells were cultured in a RAM (RAMARAJ) medium. The *Chlamydomonas reinhardtii* CC-5325 was obtained from the Chlamydomonas Resource Center at the University of Minnesota^3^ and was cultured in the TAP medium. The growth chamber was maintained at a temperature of 22 ± 1°C and a light intensity of 25 μmol photons/m^2^/s using cool-white fluorescent lamps, with a 16-h light and 8-h dark cycle.

### 2.2 Salt stress test

*Dunaliella* cultures grown to the exponential phase were inoculated into the RAM medium containing NaCl at different concentrations, including 0.5 M, 1.5 M, 2.5 M, 3.5 M, and 4.5 M, respectively. The growth curve of *Dunaliella* exposed to various salt concentrations was constructed by counting the number of cells using a cell counting chamber every 2 days. The shape, size, and color of *Dunaliella* cells were observed under the Olympus BX51 microscope.

### 2.3 Nile red staining

The Nile red dye (Shanghai Aladdin Biochemical Technology) solution at a concentration of 0.1 mg/mL was prepared with acetone as the solvent. In total, 5 mL of algal cells in the exponential phase was centrifuged at 2500 × *g* for 5 min. Discarding the supernatant, algal cells were washed twice with 1 mL of RAM medium and then resuspended with 0.2 mL of RAM medium. After adding 3 μL of Nile red dye solution to each sample, the reaction was maintained for 5 min at room temperature. The photo was recorded under the BX51 screen microscope with a 480 nm excitation wavelength. The fluorescence intensity was calculated using ImageJ software (Schneider et al., 2012).

### 2.4 Sequencing and phylogeny analysis of the *rbc*L gene

DNA was isolated with the SteadyPure Plant Genomic DNA Extraction Kit (Accurate Biotechnology). The partial *rbc*L gene of *Dunaliella* sp. FACHB-558 was amplified using PCR with specific primers (*rbc*L1-F:CGTGACAAACTAAACAAATATGG and *rbc*L1-R:AAGATTTCAACTAAAGCTGGCA), which was designed according to the conserved region of algal *rbc*Lgenes from the National Center for Biotechnology Information (NCBI). The multiple-sequence alignment of *rbc*L homologs in *Dunaliella* was analyzed, and a phylogenetic tree was constructed using MEGA 6.0 software with the maximum likelihood method using MUSCLE (MUltiple Sequence Comparison by Log-Expectation) and the Jones, Taylor, and Thorton (JTT) model with bootstrap analysis of 1,000 replicates (Tamura et al., 2013; Pan et al., 2020).

### 2.5 Inhibition test and bioremoval assays

The inhibition and bioremoval assays were performed as previously described with minor modifications (Say et al., 2003). Briefly, inductively coupled plasma mass spectrometry (ICP-MS) was used to detect the heavy metal content in the cultures every 15 days and the correction coefficient of each correction equation using standards is 0.999980 or above. The adsorption rate was calculated using the following formula,

$$\mathrm{Adsorption}=\frac{c_{0}-c_{75}}{c_{0}}$$

where c_0_ refers to the initial concentration of cobalt in the solution and c_75_ refers to the concentration of cobalt in the solution after 75 days. The standard solutions of cobalt (GBW (E)083781), nickel (GBW (E)083786), and cadmium (GBW (E)083788) were purchased from Beijing North Weiye Measurement Technology Research Institute. Cobalt chloride hexahydrate (Shanghai Macklin Biochemical Technology), nickel chloride hexahydrate (Shanghai Macklin Biochemical Technology), and cadmium chloride (Shanghai Macklin Biochemical Technology) were used as the source of heavy metal ions.

### 2.6 Growth measurement

The measurements of cell density, dry weight, and chlorophyll content were performed as described previously (Liu et al., 2023).

### 2.7 Total protein concentration

The total protein (TP) contents were measured as described previously (Liu et al., 2023). Briefly, 5 mL of algal cells was collected and mixed with Coomassie Brilliant Blue for 10 min. The optical density (OD) value at 595 nm of each sample was measured using a 1-cm optical diameter with double distilled water as the blank control. TP is calculated by the following formula,

$$TP\;\mathrm{concentration}=\frac{A_{1}-A_{CK1}}{A_{S1}-A_{CK1}}\times c_{s1}\times N$$

where $A_{1}$ refers to the OD value of the sample, $A_{CK1}$ refers to the OD value of double distilled water, $A_{S1}$ refers to the OD value of the standard, $c_{S1}$ refers to the standard concentration, and N refers to the dilution factor.

### 2.8 Determination of oxidation and antioxidative activities

The oxidation and antioxidative activities were determined according to the manufacturer’s recommended protocol. Nitric oxide (NO) induction was measured using the nitrate reductase method and the nitric oxide assay kit (Nanjing Jiancheng Bioengineering Institute). Catalase (CAT) activity was measured using the ammonium molybdate method and the catalase assay kit (Nanjing Jiancheng Bioengineering Institute). Peroxidase (POD) activity was measured using the absorbance method and the peroxidase assay kit (Nanjing Jiancheng Bioengineering Institute). Superoxide dismutase (SOD) activity was measured using the WST-1 method and the superoxide dismutase assay kit (Nanjing Jiancheng Bioengineering Institute).

### 2.9 Determination of the effects of 3-methyladenine and rapamycin

In the absorption assay, cobalt chloride was supplemented in the RAM medium at the initial inoculation. After 15 days, 300 mL of *Dunaliella* cells was subcultured into three aliquots supplemented with dissolve buffer (Mock), 3-methyladenine (3-MA), and rapamycin (RAPA), respectively. In the oxidation and antioxidation activity assays, algal cells were cultured with Mock, 3-MA, or RAPA for 1 day, and subjected to analyses.

### 2.10 Statistical analysis

The experiments were carried out with at least three biological replicates from three independent cultures, and data were reported as the mean with standard deviation (mean ± SD). Multiple comparisons were analyzed using ANOVA and Tukey’s HSD, and significant differences (*p* < 0.05) are indicated by lowercase letters. Two means were compared using Student’s *t*-test, and asterisks indicate significant differences (*p*-value <0.05). All data were analyzed using JMP Version 14 Software (SAS Institute Inc., Cary, NC).

## 3 Results and discussion

### 3.1 FACHB-558 is a salt-tolerant microalga

*Dunaliella* algae represent a genus of halophilic, unicellular, photosynthetic autotrophic microalgae known for their remarkable salt tolerance and heavy metal resistance (Borowitzka and Siva, 2007). The majority of *Dunaliella* species are indigenous to marine and saltwater environments, rendering them a promising candidate for bioremediation in marine environments. In this study, we investigated a microalga designated as FACHB-558, which was obtained from the Freshwater Algae Culture Collection at the Institute of Hydrobiology, Chinese Academy of Sciences.

**Figure 1:**
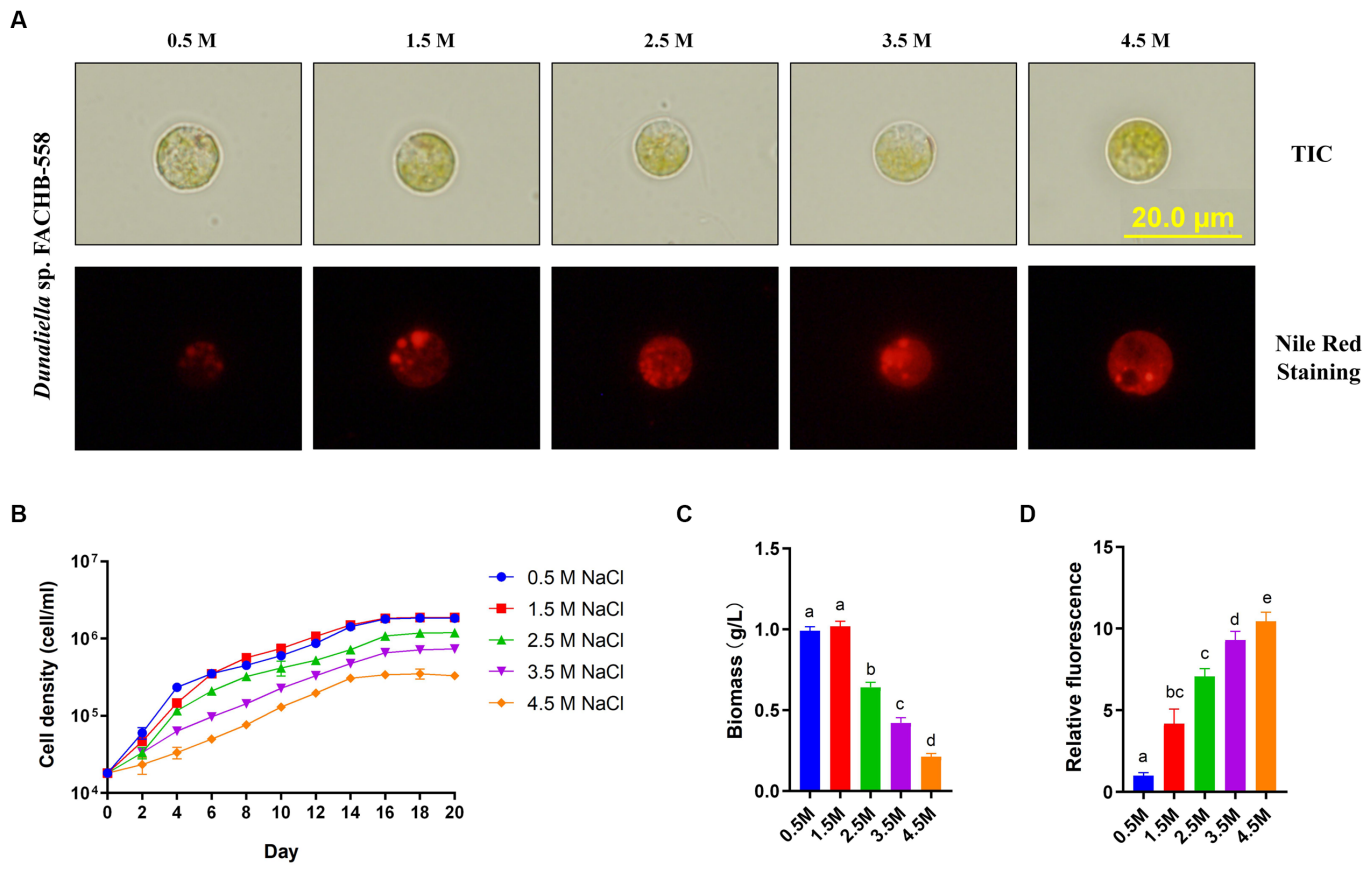
*Dunaliella* sp. FACHB-558 cultivated under different salinity conditions. **(A)** Images of *Dunaliella* sp. FACHB-558 (top) and Nile red stained cells (bottom) cultivated in RAM mediums supplemented with 0.5, 1.5, 2.5, 3.5, and 4.5 M NaCl, respectively. Scale bars = 20 μm; **(B-D)** Cell density dynamics, biomass dynamics, and quantification of fluorescence of Nile red-strained cells of *Dunaliella* sp. FACHB-558 in panel **(A)**.

Notably, a striking feature of FACHB-558 was observed in its cellular morphology grown under varied salinity conditions (Figure 1A). Unlike the classic pear-shaped morphology of typical *Dunaliella* microalgae, most FACHB-558 cells exhibited a circular shape. Our investigation of Nile red staining showed the neutral lipid content within FACHB-558 cells was increased in response to salinity stresses (Figures 1A,D), displaying a conserved salt stress response observed in various *Dunaliella* species.

Furthermore, our experiments demonstrated that FACHB-558 preferred 0.5 M NaCl and 1.5 M NaCl over other tested salinity levels (Figure 1B). This preference was corroborated by the highest biomass production under these two conditions (Figure 1C). Given that the average salinity of seawater is 3.5%, which falls within the range of 0.5 M NaCl (2.8%) to 1.5 M NaCl (8.0%), it is conceivable that the salt tolerance of FACHB-558 may have evolved as an adaptation to the marine environment.

### 3.2 Microalga FACHB-558 belongs to the genus *Dunaliella*

For several decades, the *rbc*L gene has served as a pivotal molecular marker for the study of algal diversity and phylogenetic analysis (Preetha et al., 2012; Nakada et al., 2019). The nucleotides of the partial *rbc*L gene in FACHB-558 were acquired through PCR, using designed primers based on the conserved sequences of *Dunaliella rbc*L genes available in the NCBI database. Subsequently, a phylogenetic tree encompassing a selection of previously reported *Dunaliella* microalgae was constructed using MEGA 6.0 software with the maximum likelihood method (Tamura et al., 2013).

**Figure 2:**
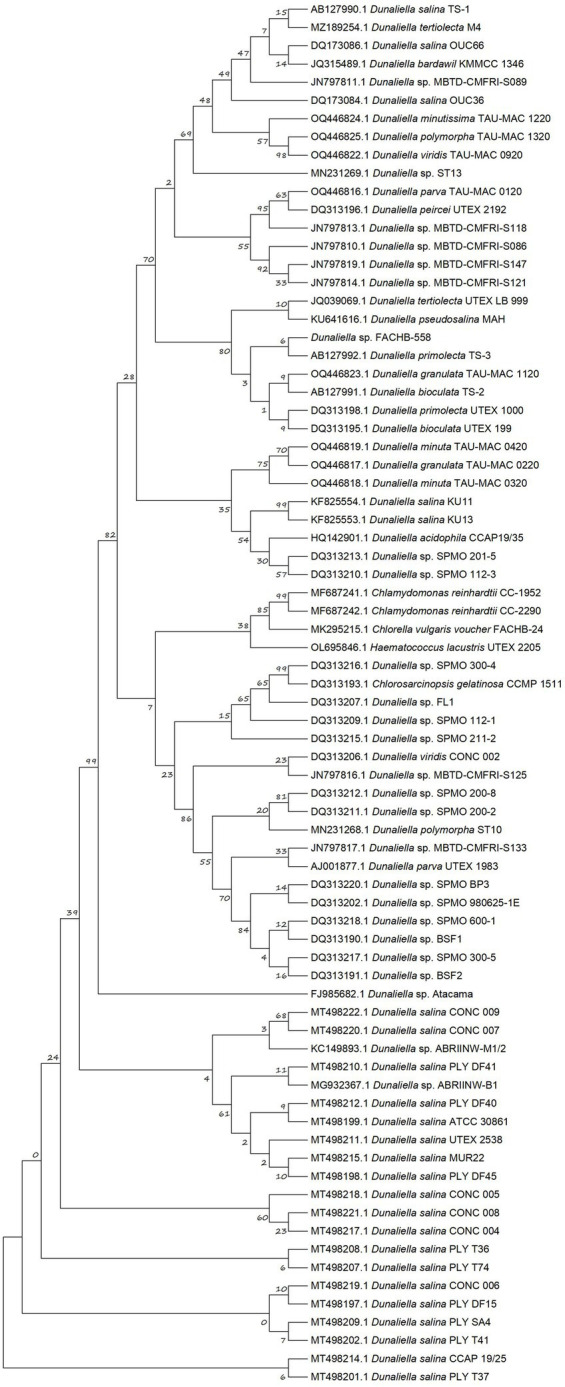
Phylogenetic tree based on partial *rbc*L sequences. The maximum likelihood (ML) phylogenetic tree was constructed from multiple-sequence alignments of *rbc*L homologs of *Dunaliella* species with MEGA6 using MUltiple Sequence Comparison by Log-Expectation (MUSCLE) and bootstrap analysis with 1,000 replicates.

As illustrated in Figure 2, the closest genetic relative of FACHB-558 is *Dunaliella primolecta* TS-3. However, the definitive classification of FACHB-558 needs further investigation at the genomic level. Notably, FACHB-558 clustered with other two branches, consisting of *D. primolecta* TS-3, *D. primolecta* UTEX 1000, *D. granulata* TAU-MAC 1120, *D. bioculata* TS-2, and UTEX 199. Together, the phylogenetic tree analysis indicated that FACHB-558 is closely related to several *Dunaliella* species, suggesting a consanguineous affiliation with *Dunaliella* species.

### 3.3 *Dunaliella* sp. FACHB-558 is tolerant of cobalt stress

**Figure 3:**
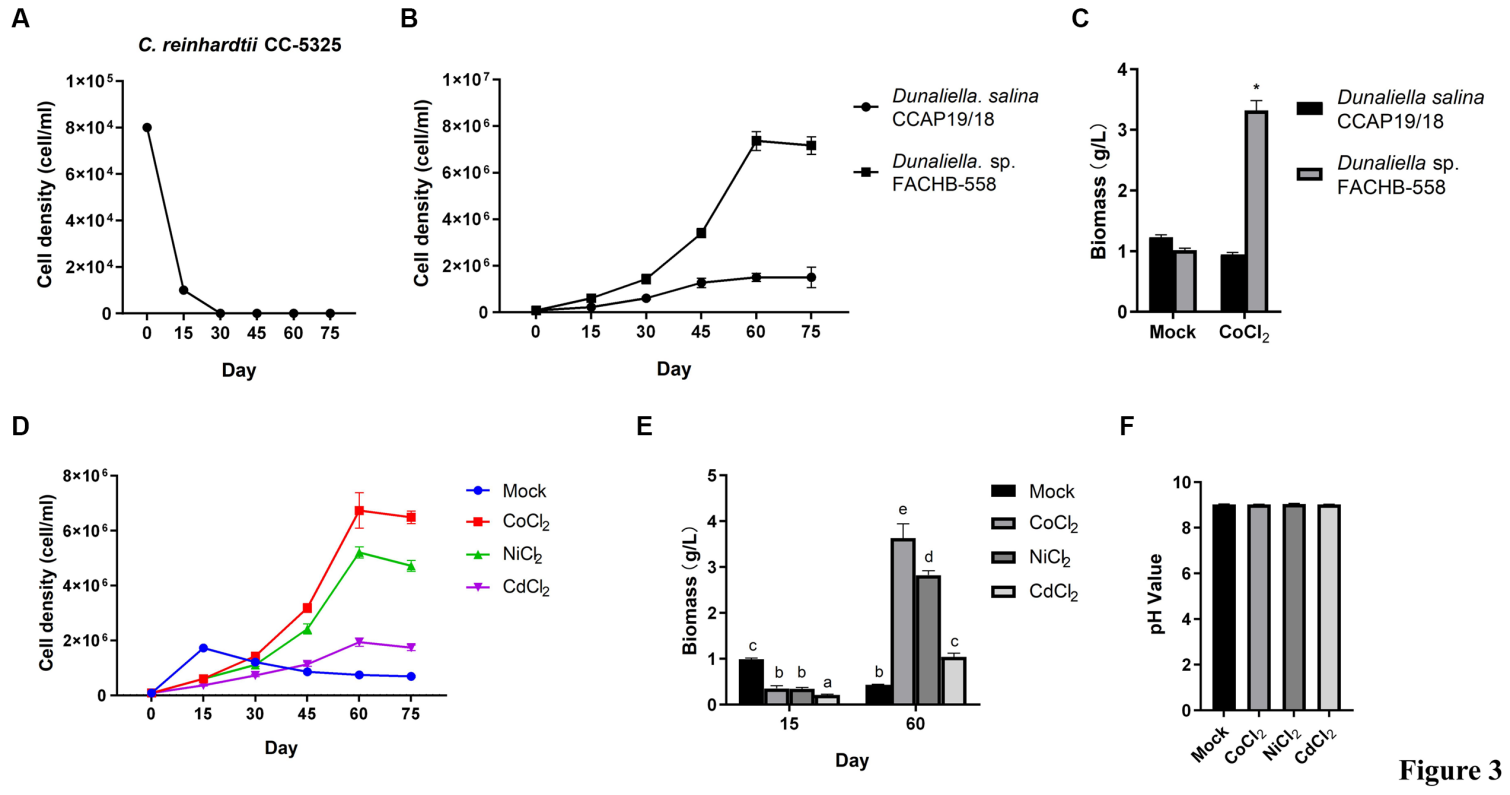
*Dunaliella* sp. FACHB-558 displayed a high tolerance to cobalt. **(A)** Cell density dynamics of *C. reinhardtii* CC-5325 cultivated in TAP medium with 60 μM CoCl_2_. **(B)** Cell density dynamics of *Dunaliella* sp. FACHB-558 and *D. salina* CCAP 19/18 cultivated in the RAM medium with 60 μM CoCl_2_. **(C)** Maximum biomass of *Dunaliella* sp. FACHB-558 and *D. salina* CCAP 19/18 cultivated by RAM medium and RAM medium with 60 μM CoCl_2_. **(D)** Cell density dynamics of *Dunaliella* sp. FACHB-558 cultivated in the RAM medium with 60 μM CoCl_2_, NiCl_2_, and CdCl_2_, respectively. **(E)** Maximum biomass of *Dunaliella* sp. FACHB-558 cultivated in the RAM medium with 60 μM CoCl_2_, NiCl_2_, and CdCl_2_ at 15 days and 60 days, respectively. **(F)**. pH values of the RAM medium were added with 60 μM CoCl_2_, NiCl_2_, and CdCl_2_ at 60 days, respectively.

In aquatic environments, cobalt mainly exists in the form of Co^2+^ or Co^3+^ (Jiang et al., 2022). In order to test the toxicity of Co^2+^ on microalgae, the cell density of *Dunaliella* sp. FACHB-558 grown 75 days was measured in RAM media supplemented with 60 μM cobalt chloride. Two other microalgae, *Dunaliella salina* CCAP 19/18 and *Chlamydomonas reinhardtii* CC-5325, were used in this assay as controls, representing the species of *Dunaliella* and green algae, respectively. As shown in Figure 3A, *C. reinhardtii* CC-5325 displayed retarded growth in the first 15 days, and followed by cell death over approximately 30 days. Interestingly, *Dunaliella* species kept growing from 30 days to 75 days and reached the stationary phase at approximately 60 days, although the population density of *Dunaliella salina* CCAP 19/18 was much lower than that of *Dunaliella* sp. FACHB-558 (Figure 3B). As we expected, *Dunaliella* sp. FACHB-558 in the Mock (RAM media only) with cobalt was much higher than that in RAM without cobalt, while the maximum biomass of CCAP 19/18 was almost unaffected by cobalt (Figure 3C). Together, these results showed that 60 μM cobalt was detrimental to other green algae such as *C. reinhardtii* CC-5325, toxic to *D. salina* CCAP 19/18, but beneficial to *Dunaliella* sp. FACHB-558.

In addition to cobalt, the effects of two other heavy metals, including nickel and cadmium, on the growth of *Dunaliella* sp. FACHB-558 were determined. Cadmium stress suppressed the growth of *Dunaliella* sp. FACHB-558 (Figure 3D), while nickel promoted its growth in a similar way as cobalt did, although the maximum cell density in nickel treatment was slightly lower than that in cobalt treatment (Figure 3D). The biomass of FACHB-558 grown under different heavy metal treatments displayed consistent trends, as observed in cell density dynamics (Figure 3E). The biomass of FACHB-558 grown without stress (the Mock) was highest at 15 days. Its quality was sorted as cobalt > nickel > cadmium > RAM when grown under stress for 60 days. Notably, the cell density under cobalt treatment or nickel treatment was approximately three times that in the Mock, indicating that *Dunaliella* sp. FACHB-558 can effectively metabolize these metals.

The trace cobalt, mainly existing in vitamin B12, was identified as an essential element for the growth of *D. tertiolecta* (Chen et al., 2011). In a chronic toxicity test on 10 marine species, *D. tertiolecta* exhibited the second-lowest sensitivity toward cobalt treatment (Saili et al., 2021). In the classical RAM medium designed for *Dunaliella* algae, the working concentration of CoCl₂·6H₂O is 0.21 μM (Wu et al., 2017). In this study, it was found that *D. salina* CCAP 19/18 showed delayed growth rates under 60 μM cobalt, suggesting toxicity of cobalt on *Dunaliella* algae (Figure 3B). However, *Dunaliella* sp. FACHB-558 maintained an increased cell density under 60 μM cobalt, indicating the promotion effects of cobalt on some *Dunaliella* algae (Figures 3B,D). On the other hand, *D. salina* CCAP 19/18 could survive under 60 μM cobalt, which was detrimental to the green alga *C. reinhardtii* (Figure 3A). In future research, it would be interesting to investigate how *Dunaliella* sp. FACHB-558 responds to lower and higher concentrations of cobalt ions and to compare its performance with that of other microalgae species, not limited to *C. reinhardtii*. Moreover, *Dunaliella* sp. FACHB-558 was tolerant to 60 μM cadmium and favored 60 μM nickel. This specificity is not related to the pH value in the medium, because the determination of the pH value of the medium showed there were no significant differences among heavy metal treatments (Figure 3F).

### 3.4 *Dunaliella* sp. FACHB-558 could remove cobalt from saltwater

**Figure 4:**
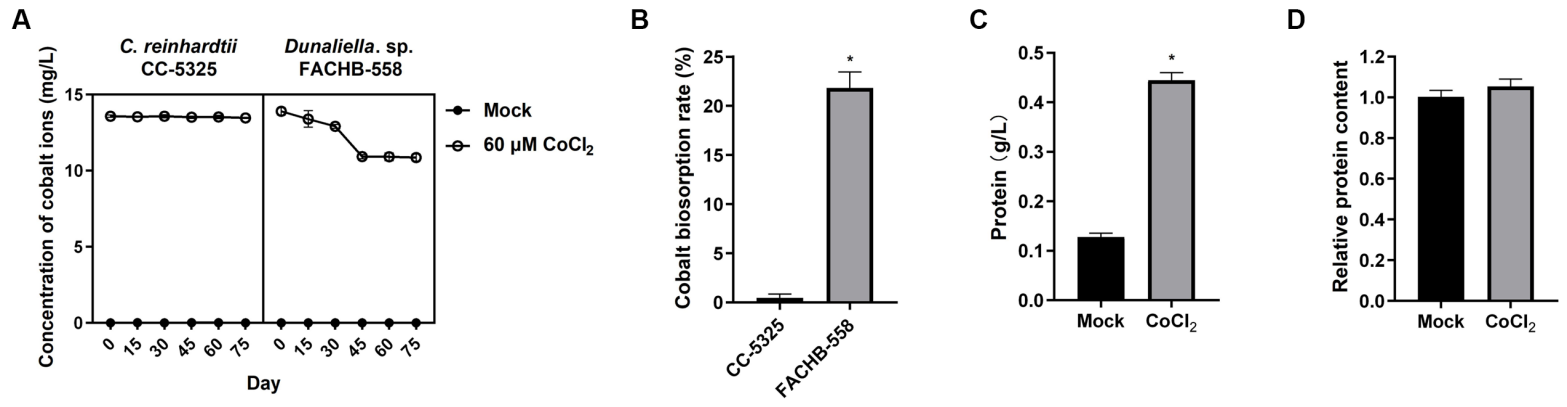
*Dunaliella* sp. FACHB-558 possesses a higher cobalt biosorption capacity. **(A)**. Concentrations of cobalt ions in the medium cultured with *C. reinhardtii* CC-5325 and *Dunaliella* sp. FACHB-558, respectively. **(B)**. Absorption rate of cobalt ions by *C. reinhardtii* CC-5325 and *Dunaliella* sp. FACHB-558, respectively. **(C)**. Total protein yield per liter of *Dunaliella* sp. FACHB-558 cultivated by RAM mediums and RAM medium with 60 μM CoCl_2_. **(D)**. Total protein content per cell of *Dunaliella* sp. FACHB-558 cultivated in the RAM medium and RAM medium with 60 μM CoCl_2_.

To figure out if the high tolerance of *Dunaliella* sp. FACHB-558 to heavy metal ions resulted from its high metal consumption, the concentration of cobalt in *Dunaliella* sp. FACHB-558-containing media was measured with *C. reinhardtii* as control. As shown in Figure 4A, the concentration of cobalt in the *C. reinhardtii*-containing media barely changed within 75 days, suggesting its inability to remove the cobalt as a natural biosorbent, probably due to its limited biomass and low survival rate under cobalt stress (Figure 3A). In contrast, the cobalt concentration gradually decreased in *Dunaliella* sp. FACHB-558-containing media, with a sharp drop from 30 days to 45 days at the early exponential growth phase (Figures 3B, 4A). In line with this, the quantified biosorption rate showed *Dunaliella* sp. FACHB-558 could remove approximately 21.8% cobalt from saltwater (Figure 4B). Moreover, the cobalt biosorption by *Dunaliella* sp. FACHB-558 did not alter relative protein content per cell (Figure 4D) but increased the TP yield via the promotion of the cell density of *Dunaliella* sp. FACHB-558 (Figures 3D, 4C).

Two *Dunaliella* species used in this study had much higher tolerance to different concentrations of cobalt than *C. reinhardtii*, suggesting the advantages of *Dunaliella* microalgae for heavy metal bioremediation. Despite the fact that it takes 45 days for *Dunaliella* sp. FACHB-558 to reduce cobalt concentration from 14 mg/L to approximately 10 mg/L, we demonstrated it is a halotolerant microalgae strain functioning as a live biosorbent in saltwater and other hypersaline environments.

### 3.5 *Dunaliella* sp. FACHB-558 exhibited enhanced NO production under cobalt stress

**Figure 5:**
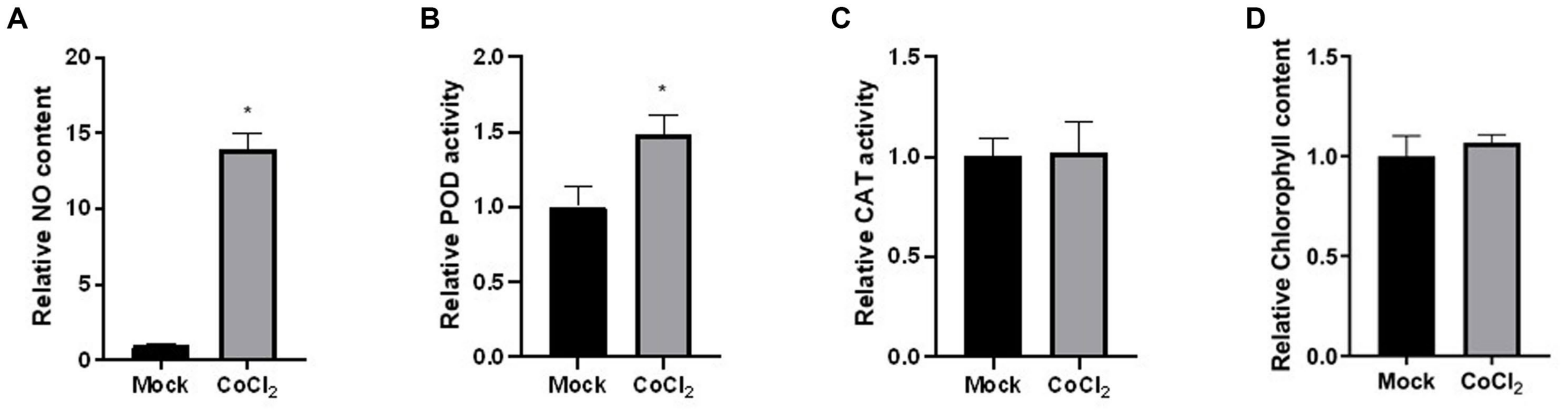
Physiological and biochemical responses of *Dunaliella* sp. FACHB-558 under the cobalt treatment. NO content **(A)**, POD enzymatic activity **(B)**, CAT enzymatic activity **(C)**, and chlorophyll content **(D)** per cell of *Dunaliella* sp. FACHB-558 cultivated in the RAM medium and RAM medium with 60 μM CoCl_2_.

Reactive oxygen-derived free radicals, such as superoxide radical, hydroxyl radical, and hydrogen peroxide, are known to be produced in algae under heavy metal stress (Leong and Chang, 2020). This oxidative stress leads to membrane lipid peroxidation, DNA and protein denaturation, enzyme activity reduction, membrane system damage, and cell apoptosis (Haeder and Gao, 2015; Garcia-Garcia et al., 2016). In response to the toxic effects of heavy metals, algae have evolved antioxidant enzymes, including CAT, POD, and SOD. While SOD activity in cells of *Dunaliella* sp. FACHB-558 could not be successfully detected, the assays on CAT activity and POD activity provide insights into the biochemical responses of algal cells under severe cobalt stress (Figures 5B,C). Given the influence of cobalt stress on the biomass or the cell density of *Dunaliella* sp. FACHB-558 (Figure 3D), our focus was directed toward measuring enzyme activity per cell rather than per liter. The CAT activity per cell of *Dunaliella* sp. FACHB-558 remained relatively unchanged with the application of cobalt (Figure 5C), whereas a slight increase in POD activity was observed (Figure 5B). Interestingly, chlorophyll content per cell was not affected by excess cobalt in RAM medium (Figure 5D). In future studies, significant attention could be devoted to elucidating the role of POD in the cellular responses of *Dunaliella* algae to heavy metals. Furthermore, it appeared that cobalt stress did not induce significant oxidation damage in the cells of *Dunaliella* sp. FACHB-558. However, further investigations are warranted to quantify the intracellular and extracellular ROS production under heavy metal stresses.

Interestingly, our results indicated an increase in NO production in *Dunaliella* sp. FACHB-558 under severe cobalt stress (Figure 5A). In diverse physiological processes in algae and high plants, NO has been found to have both beneficial and detrimental effects (Galatro et al., 2020; Kuo et al., 2020). Plants produce NO in response to abiotic stresses and utilize it to alleviate the damage caused by ROS (Qiao and Fan, 2008). As a redox-related signaling molecule, NO is implicated in post-translational modifications such as S-nitrosylation of cysteine, and nitration of tyrosine and tryptophan (Leon, 2022). In the context of cobalt-stressed *Dunaliella* sp. FACHB-558, we speculate that excess heavy metal induces a burst of reactive oxygen species (ROS) and NO acts to relieve the oxidative damage in algae, which is similar to that in plants (Wei et al., 2020). Nevertheless, additional research is needed to elucidate the causal relationship between NO production and cobalt tolerance in *Dunaliella* microalgae.

### 3.6 Autophagy negatively regulates the tolerance of FACHB-558 to cobalt

Autophagy, a crucial physiological process in eukaryotes, plays a pivotal role in numerous pathological and physiological contexts by facilitating the degradation of misfolded proteins and abnormal organelles (Mizushima and Komatsu, 2011). This cellular mechanism not only supplies essential raw materials and energy for various cellular activities but also promotes cell survival. However, excessive autophagy can trigger programmed cell death (Li et al., 2020). Consequently, autophagy inhibitors and activators have become valuable tools in the functional investigation of autophagy processes (Sheng et al., 2010; Kim et al., 2011; Yang et al., 2020). For example, 3-methyladenine (3-MA) is a well-established autophagy inhibitor that exerts its effects by targeting PI3K, and recent studies have revealed its ability to modulate autophagy even in unicellular algae (Sheng et al., 2010). Conversely, RAPA acts by inhibiting the mammalian target of the RAPA (mTOR) pathway and thereby activating autophagy (Deleyto-Seldas and Efeyan, 2021).

**Figure 6:**
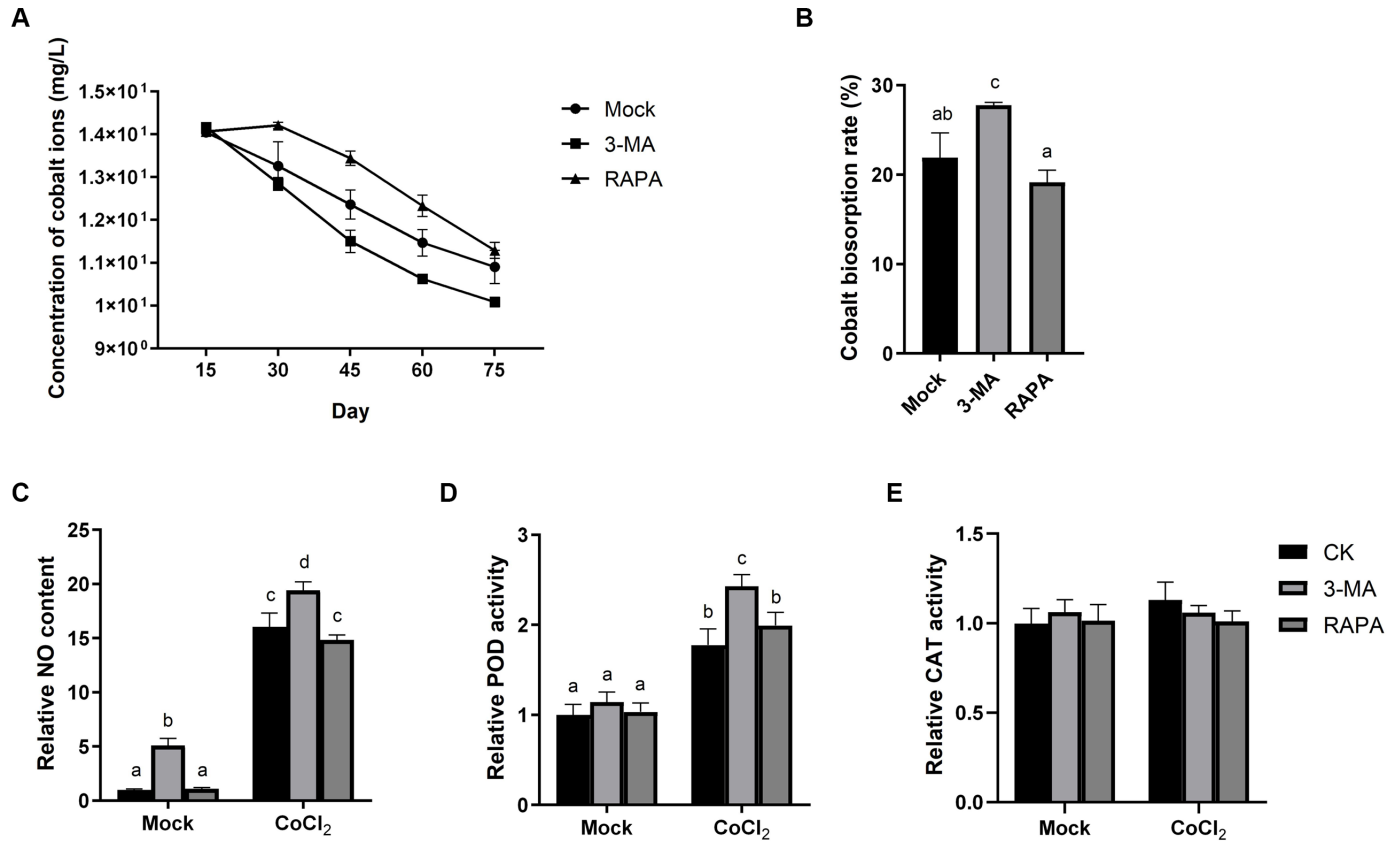
Autophagy negatively regulates the tolerance of *Dunaliella* sp. FACHB-558 to cobalt stress. **(A)** Concentrations of cobalt ions in the RAM medium cultured with *Dunaliella* sp. FACHB-558. Autophagy inhibitor 3-MA and autophagy activator RAPA were applied, respectively. **(B)** Absorption rate of cobalt ions by *Dunaliella* sp. FACHB-558 under treatments of 3-MA and RAPA. NO content **(C)**, POD enzymatic activity **(D)**, and CAT enzymatic activity **(E)** per cell of *Dunaliella* sp. FACHB-558 cultivated in the RAM medium and RAM medium with 60 μM CoCl_2_.

To elucidate the role of autophagy in the cobalt tolerance of *Dunaliella* sp. FACHB-558, we applied -3-MA and RAPA to algal cells grown under severe cobalt stress. The biosorption assay demonstrated that 3-MA accelerated the bioremoval of cobalt, while RAPA decelerated this process (Figure 6A), indicating that autophagy negatively impacts the removal of cobalt ions. Furthermore, the cobalt biosorption rate increased significantly with the application of 3-MA, although no significant alternation was observed with the application of RAPA (Figure 6B).

Interestingly, 3-MA was found to enhance NO production per cell and increase POD activity per cell, both in the presence and absence of cobalt (Figures 6C,D). This suggests that autophagy negatively regulates the NO content in algal cells. In contrast, our results indicated that RAPA has no significant impact on NO production, POD activity, or CAT activity (Figures 6C–E). Considering the observed negative effect of RAPA on cobalt biosorption by *Dunaliella* sp. FACHB-558 (Figure 6A), we speculated that the activation of autophagy did not alter the oxidation status in algal cells. However, we cannot rule out the possibility that RAPA may have additional targets in algal cells aside from TOR.

In summary, the use of the autophagy inhibitor 3-MA resulted in increased NO production and POD activity in *Dunaliella* sp. FACHB-558 cells, ultimately enhancing the efficiency of cobalt removal from saltwater. Future studies aiming to decipher the underlying mechanism of how autophagy regulates cobalt bioremoval will provide insights into optimizing natural algae-based biosorption processes.

## 4 Conclusion

Microalgae-based heavy metal biosorption technology offers substantial and sustainable advantages, including energy efficiency and carbon sequestration capabilities (Kumar et al., 2021). This study focuses on the identification of the novel *Dunaliella* strain FACHB-558, characterizing its responses to 60 μM cobalt and unraveling the mechanism of its impressive tolerance to heavy metal stress. Our findings highlight the exceptional resilience of this halotolerant microalgae to cobalt stress and its capacity for effective cobalt ion absorption.

Notably, our results reveal the involvement of POD activity, NO production, and autophagy in regulating the tolerance of *Dunaliella* sp. FACHB-558 to heavy metal stresses. These insights into the molecular and physiological responses provide a deeper understanding of the microalgae’s remarkable adaptability.

Overall, our study presents *Dunaliella* sp. FACHB-558 as a promising and natural biosorbent for mitigating cobalt pollution in saltwater and seawater environments. This research contributes to the development of sustainable and environmentally friendly solutions for addressing heavy metal contamination, aligning with the principles of energy efficiency and carbon sequestration in microalgae-based bioremediation.

## Data availability statement

The original contributions presented in the study are included in the article/Supplementary material. Further inquiries can be directed to the corresponding author.

## Author contributions

CL: Conceptualization, Data curation, Formal analysis, Investigation, Methodology, Visualization, Writing – original draft, Writing – review & editing. XW: Formal analysis, Investigation, Methodology, Writing – review & editing. HP: Formal analysis, Writing – review & editing. YL: Formal analysis, Investigation, Writing – review & editing. JZ: Investigation, Writing – review & editing. YW: Investigation, Writing – review & editing. ZZ: Resources, Writing – review & editing. TS: Methodology, Writing – review & editing. JC: Methodology, Writing – review & editing. ZH: Supervision, Writing – review & editing. SL: Funding acquisition, Project administration, Supervision, Writing – review & editing. HL: Conceptualization, Funding acquisition, Project administration, Supervision, Writing – review & editing.

## Footnotes

^1^ http://algae.ihb.ac.cn/English/

^2^ http://www.ccap.ac.uk/

^3^ https://www.chlamylibrary.org/
